# Biomarker study of pembrolizumab in patients with advanced rare cancers

**DOI:** 10.1016/j.xcrm.2026.102827

**Published:** 2026-06-16

**Authors:** Aung Naing, Xiqi Li, Qi Wang, Xiangjun (Jun) Tian, Sharjeel Sabir, Priya R. Bhosale, Mohamed H. Derbala, Bettzy Stephen, Mingxuan Xu, Joud Hajjar, Serdar A. Gurses, Yali Yang, Hassan Ahmed Momin, Ignacio Ivan Wistuba, Anas Alshawa, Kenna R. Shaw, Abdulrazzak Zarifa, Gopal Singh, Renganayaki Krishna Pandurengan, Gabriela Maria Raso, Edwin Roger Parra, Juhee Song, Mohammad Moustaf Mohammad, Jordi Rodon Ahnert, Siqing Fu, Vivek Subbiah, Sarina Anne Piha-Paul, Jing Wang, Scott Eric Woodman, Funda Meric-Bernstam

**Affiliations:** 1 Investigational Cancer Therapeutics, The University of Texas MD Anderson Cancer Center, Houston, TX 77030, USA; 2 Genomic Medicine, The University of Texas MD Anderson Cancer Center, Houston, TX 77030, USA; 3 Bioinformatics & Comp Biology, The University of Texas MD Anderson Cancer Center, Houston, TX 77030, USA; 4 Interventional Radiology, The University of Texas MD Anderson Cancer Center, Houston, TX 77030, USA; 5 Abdominal Imaging, The University of Texas MD Anderson Cancer Center, Houston, TX 77030, USA; 6 Section of Immunology, Allergy and Rheumatology, Baylor College of Medicine and Texas Children’s Hospital, Houston, TX 77030, USA; 7 Translational Molecular Pathology, The University of Texas MD Anderson Cancer Center, Houston, TX 77030, USA; 8 Institute for Personalized Cancer Therapy, The University of Texas MD Anderson Cancer Center, Houston, TX 77030, USA; 9 Biostatistics, The University of Texas MD Anderson Cancer Center, Houston, TX 77030, USA

**Keywords:** rare cancer, pembrolizumab, biomarker, tumor microenvironment, immune infiltration, spatial immune context

## Abstract

Rare cancers lack robust, evidence-based treatment options. We conduct a prospective, multi-histology phase 2 clinical trial of pembrolizumab in 154 patients (142 evaluable), observing an objective response rate of 14.8% and clinical benefit (CB) in 26.8%. Multi-modal profiling is performed on baseline, on-treatment, and progression samples. CB associates with high microsatellite instability (MSI-H)/high tumor mutation burden (TMB-H) (odds ratio [OR]: 13.9, *p* = 0.0013) and programmed cell death ligand 1 (PD-L1) combined positive score (CPS) ≥10 (*p* = 0.0285), although responses also occur in biomarker-negative tumors and vary by histology. CB tumors exhibit higher pre-treatment immune infiltration and T cell activation, whereas no CB (NCB) tumors show proliferative programs. In moderately infiltrated tumors, CB associates with increased immune content during treatment. Multiplex immunofluorescence confirms higher baseline T cell densities in CB and limited remodeling in NCB tumors. These findings suggest that tumor immune microenvironment features may serve as predictive markers beyond genomic assays and highlight immune cell recruitment during therapy in moderately infiltrated tumors.

## Highlights

• Pembrolizumab achieved clinical benefit across multiple advanced rare cancer types
• MSI-H/TMB-H enrich for response but explain only a small subset of clinical benefit
• Intermediate infiltration tumors respond via treatment-induced immune activation
• Multi-model profiling reveals immune contexture as a key determinant of response

## Graphical abstract

**Figure:**
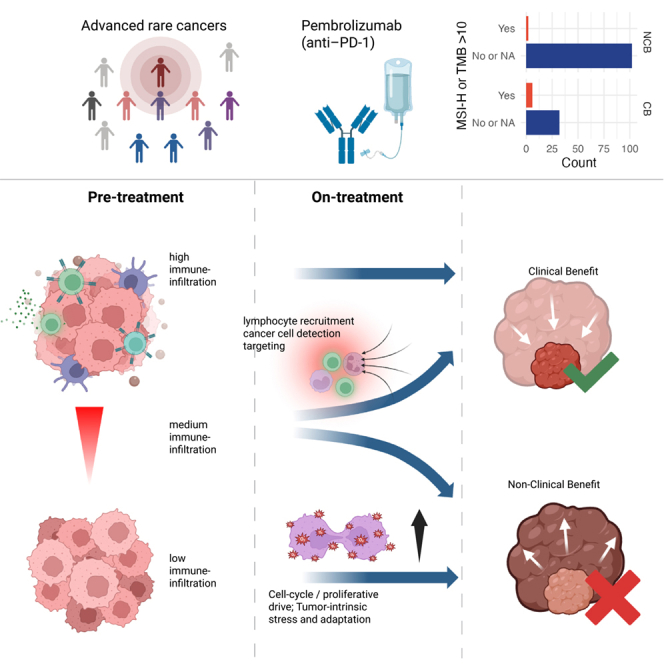

## Teaser

Naing et al. study pembrolizumab in advanced rare cancers and show that immune contexture, rather than genomic biomarkers alone, associates with clinical benefit, with responses enriched by MSI-H/TMB-H and driven by pre-existing or treatment-induced immune activation in moderately infiltrated tumors.

## Introduction

Rare cancers are a heterogeneous group of cancers, defined by the US National Cancer Institute as those with an incidence of less than 15 cases per 100,000 people per year.^1^ Despite accounting for approximately 25% of cancer-related deaths in the United States, rare cancers remain understudied, with few evidence-based treatment options—largely due to substantial barriers in clinical trial accrual.

In recent years, modulation of immune inhibitory pathways using immune checkpoint inhibitors (ICIs) has emerged as the new cornerstone for anticancer treatment.^2^ Pembrolizumab, a programmed cell death 1 (PD-1) inhibitor, has been approved by the US Food and Drug Administration (FDA) for the treatment of multiple cancers,^3^ including tumors with high microsatellite instability (MSI-H) or mismatch repair deficiency^4^ and high tumor mutation burden (TMB-H).^5^ Its tumor-agnostic approvals have created a unique opportunity to evaluate immune checkpoint blockade in historically understudied populations.

Leveraging the rare cancer patient volume at The University of Texas MD Anderson Cancer Center, we conducted an investigator-initiated, prospective, multi-histology, phase 2 basket trial (ClinicalTrials.gov number: NCT02721732) to evaluate pembrolizumab in patients with advanced rare cancers.^6^ In parallel, we integrated a comprehensive translational research component, performing biomarker analyses on baseline and on-treatment paired tumor samples to elucidate molecular and immune correlates of response. Notably, a subset of patients exhibited an objective response or durable clinical benefit (CB). While some of these responses aligned with known biomarkers of ICI sensitivity—including programmed cell death ligand 1 (PD-L1) expression,^7^^,^^8^ MSI-H status,^4^ and TMB-H status,^5^ factors typically associated with tumor response—others occurred in the absence of such biomarkers, underscoring the limitations of current predictive markers and the critical need to identify other biomarkers of response.

Improved biomarker-driven patient selection is essential to maximizing the CB of ICIs while minimizing the risk of immune-related adverse events and the substantial cost of immunotherapy. Herein, we report the results of an in-depth biomarker analysis incorporating paired baseline and on-treatment tumor samples, with the goal of uncovering molecular and immune correlates of response to PD-1 blockade in advanced rare cancers.

## Results

### Patient cohort and association of MSI-H/TMB-H and PD-L1 with CB

A total of 154 patients with advanced rare cancers treated with pembrolizumab between August 25, 2016, and August 8, 2018, were included in the current study. The baseline patient characteristics are provided in Table 1. Of the 154 patients, 142 were evaluable for response. Twelve patients were not evaluable for response for the following reasons: stopped treatment due to toxicity (*n* = 4), withdrew consent (*n* = 4), Principal Investigator decision (*n* = 1), patient choice (*n* = 1), or response not evaluable by date of data cutoff (*n* = 2). Of the 142 patients evaluable for response, 21 had an objective response, for an objective response rate (ORR) of 14.8%, and 38 patients (26.8%) achieved CB. Except for patients harboring small-cell malignancies of non-pulmonary origin (*n* = 12) or medullary renal cell carcinoma (*n* = 5), CB was observed in each of the other rare cancer types (Table S1). Patient-level response patterns and treatment duration across tumor types are shown in Figures S1 and S2.

**Table 1:** Baseline patient characteristics (*n* = 154)

Characteristic	No. (%)
Age, years	
Median	57
Range	22–88
Gender	
Male	81 (52.6)
Female	73 (47.4)
Race	
White or Caucasian	116 (75.3)
Black or African American	13 (8.4)
Other	15 (9.7)
Unknown	2 (1.3)
Ethnicity	
Not Hispanic or Latino	136 (88.3)
Hispanic or Latino	15 (9.7)
Declined to answer	1 (0.6)
Unknown	2 (1.3)
No. of prior therapies	
Median	2
Range	0–10
Cohorts	
1. Squamous cell carcinoma of the skin^a^	20 (13.0)
2. Small-cell malignancies of non-pulmonary origin	12 (7.8)
3. Adrenocortical carcinoma	22 (14.3)
4. Medullary renal cell carcinoma	5 (3.2)
5. Carcinoma of unknown primary	29 (18.8)
6. Penile carcinoma	3 (1.9)
7. Vascular sarcoma	11 (7.1)
8. Germ cell/testicular tumor	12 (7.8)
9. Paraganglioma/pheochromocytoma	12 (7.8)
10. Other rare histologies	28 (18.2)
ECOG performance status	
0	17 (11.0)
1	137 (89.0)
PD-L1 expression	
CPS <10	86 (55.8)
CPS ≥10	48 (31.2)
Missing	20 (13.0)
TIL score	
0	5 (3.2)
1	60 (39.0)
2	34 (22.1)
3	42 (27.3)
Missing	13 (8.4)

CPS, combined positive score; ECOG, Eastern Cooperative Oncology Group; PD-L1, programmed cell death ligand 1; TIL, tumor-infiltrating lymphocyte.

See also Figures S1 and S2; Table S1.

a All individuals in this cohort have metastatic disease.

**Figure 1:**
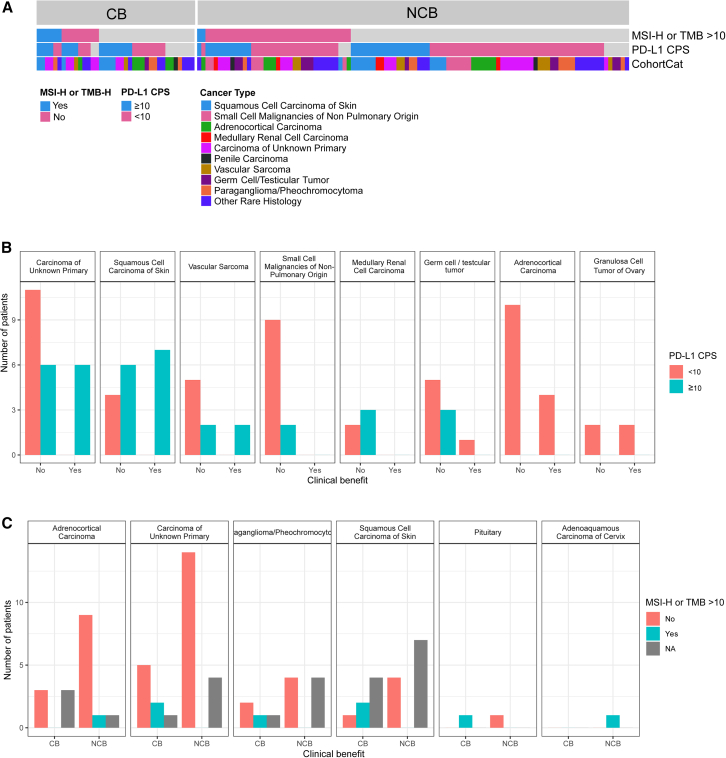
Effect of factors known to be associated with pembrolizumab response (A) Clinical benefit (CB) and no clinical benefit (NCB) among patients stratified by high microsatellite instability (MSI-H) or high tumor mutation burden status (TMB-H; TMB > 10) and PD-L1 combined positive score (CPS) <10 or ≥10, shown by tumor type. See also Table S2. (B) Number of patients with CB or NCB in representative rare tumor groups, by PD-L1 status (CPS < 10 or ≥ 10). (C) Number of patients with CB or NCB in selected rare tumor groups in which MSI-H or TMB-H was observed, stratified by MSI-H or TMB-H status.

MSI-H^4^ and TMB-H^5^ have both been shown to be associated with the clinical efficacy of immune checkpoint blockade agents. In the current study, among the tumor samples in which levels of these biomarkers were tested, 8.8% (5 of 57) had MSI-H and 6.2% (6 of 97) had TMB-H (i.e., >10 mutations per megabase). When evaluating the relationship between these biomarkers and clinical outcome, we observed that 26.1% (6 of 23) of patients who achieved CB had MSI-H or TMB-H, compared with only 2.5% (2 of 81) of patients with no CB (NCB; odds ratio [OR]: 13.9, 95% confidence interval [CI]: 2.6–75.1; *p* = 0.0013, Fisher’s exact test; Table S2; Figure 1A).

To evaluate the association of PD-L1 with clinical outcome, we determined the global PD-L1 content within 124 of 142 pre-treatment samples in our cohort. A combined positive score cutoff level as low as 10 was significantly associated with higher odds of having CB (PD-L1 ≥10, OR: 2.5, 95% CI: 1.1–5.9; *p* = 0.0285, chi-square test; Figure 1A). However, this association was not consistent across all rare tumor types. Among patients with carcinoma of unknown primary, squamous cell carcinoma of the skin, or vascular sarcoma, only those with a pre-treatment PD-L1 combined positive score ≥10 achieved a CB (carcinoma of unknown primary: 26% [6 of 23]; squamous cell carcinoma of the skin: 41% [7 of 17]; vascular sarcoma: 22% [2 out of 9]; Figure 1B). For other rare cancer types, the relationship was less consistent. All patients with small-cell malignancies of non-pulmonary origin and medullary renal cell carcinoma and 8 out of 9 patients with germ cell/testicular tumors had NCB, regardless of PD-L1 levels. In contrast, 28.6% (4 of 14) of patients with adrenocortical carcinoma and 50.0% (2 of 4) of patients with granulosa cell tumors (included in the “other rare histologic types” group) achieved CB, despite all having a PD-L1 combined positive score <10. Analogous to PD-L1 combined positive score, the association between MSI-H/TMB-H and CB was heterogeneous across tumor types (Figure 1C). MSI-H/TMB-H was more frequently observed among patients with CB in select histologies, including carcinoma of unknown primary and squamous cell carcinoma of the skin; however, CB was also observed in the absence of MSI-H/TMB-H. MSI-H/TMB-H did not uniformly correspond to benefit across tumor histologies.

### Baseline transcriptomic landscape of CB tumors versus NCB tumors

**Figure 2:**
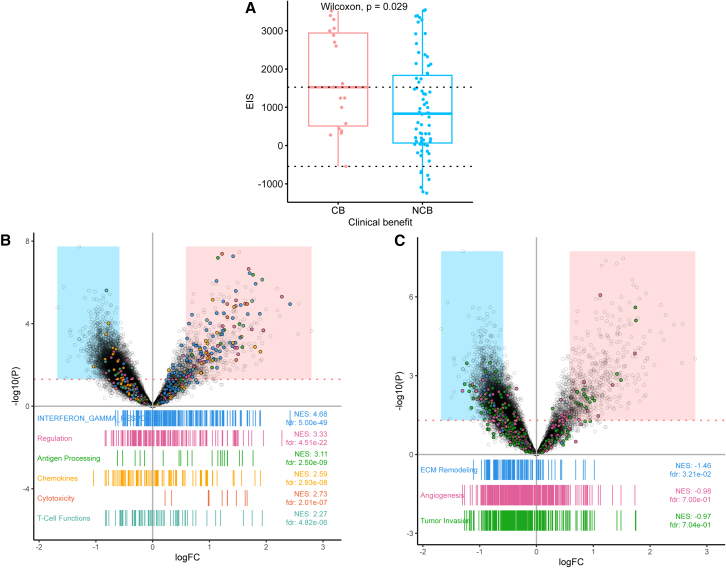
Immune infiltration and gene transcription programs in pre-treatment tumors from patients who had a CB or NCB from pembrolizumab (A) ESTIMATE immune score (EIS) in patients with CB (*n* = 19) and NCB (*n* = 70). *p* value (Wilcoxon rank-sum test) is shown for between-group comparisons. (B and C) Differential gene expressions and gene transcription programs associated with CB (B) and NCB (C). 645 CB-associated and 3,125 NCB-associated genes (|log2 fold change| > log2(1.5), *p* value < 0.05) are indicated by red and blue shaded rectangles, respectively. Enrichment of gene sets with normalized enrichment score (NES) and *q* value is shown below. ECM, extracellular matrix. See also Figure S3.

To interrogate the immune landscape of tumors associated with CB or NCB of pembrolizumab, we performed transcriptomic analysis on pre-treatment samples (*n* = 89). We calculated an immune score to globally characterize the immune status of each tumor sample.^9^ Patients who achieved CB displayed greater overall immune content in their pre-treatment tumors compared with NCB patients (*p* = 0.029, Wilcoxon rank-sum test; Figure 2A). Bootstrap resampling confirmed consistent directional differences, with higher pre-treatment immune scores in CB observed in 95.2% of resampled datasets (Figure S3A). In mixed-effects logistic regression accounting for tumor type, higher immune scores remained significantly associated with CB (OR: 1.76 per SD; *p* = 0.039), and leave-one-tumor-type-out analyses showed stable effect directionality (Figure S3B). To explore the gene expression programs underlying tumors associated with CB or NCB, we performed gene set enrichment analysis on the most differential genes between these outcomes. The pre-treatment tumors of patients who achieved CB were enriched with multiple transcripts associated with interferon-gamma (IFN-γ), T cell function, regulation, cytotoxicity, chemokine/cytokine levels, and antigen processing (Figure 2B). Conversely, the tumors of NCB patients were enriched with transcripts associated with tumor invasion, extracellular matrix remodeling, and angiogenesis (Figure 2C).

### Divergent immune and proliferative dynamics in on-treatment tumors

**Figure 3:**
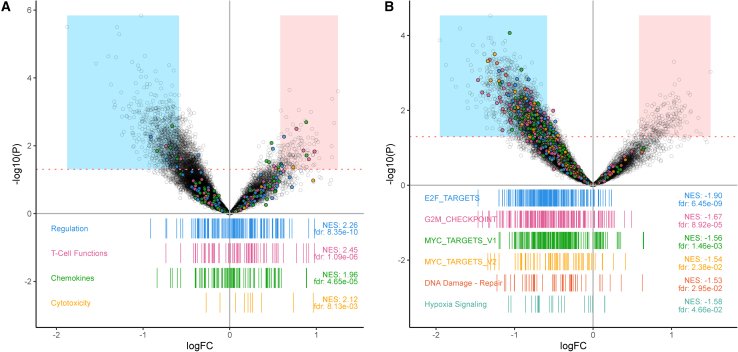
Treatment-induced alterations in gene expression levels associated with CB (*n* = 15) or NCB (*n* = 56) (A) Upregulated gene transcription programs associated with CB. (B) Upregulated gene transcription programs associated with NCB. Differential gene expression analysis was performed on the delta (on-treatment − pre-treatment) expression profiles. 152 CB-associated and 966 NCB-associated genes (|log2 fold change| > log2(1.5), *p* value < 0.05) are indicated by red and blue shaded rectangles, respectively. Enrichment of gene sets with normalized enrichment score (NES) and *q* value is shown below.

In addition to pre-treatment samples (*n* = 89), matched on-treatment tumor samples were available in a subset of patients (*n* = 71). In most cases, a lesion selection tool was applied to sample the same area consistently. The gene transcripts displaying the greatest increase within on-treatment tumors of patients achieving CB were associated with many of the same immune features observed to be enriched in the pre-treatment tumors of patients with CB, namely, T cell function, regulation, cytotoxicity, and chemokines (Figure 3A), whereas on-treatment tumors of patients with NCB were enriched in genes associated with cell cycle regulation (e.g., E2F, G2M, MYC, hypoxia, and DNA damage repair; Figure 3B).

### Baseline immune infiltration and treatment-induced immune remodeling in CB

**Figure 4:**
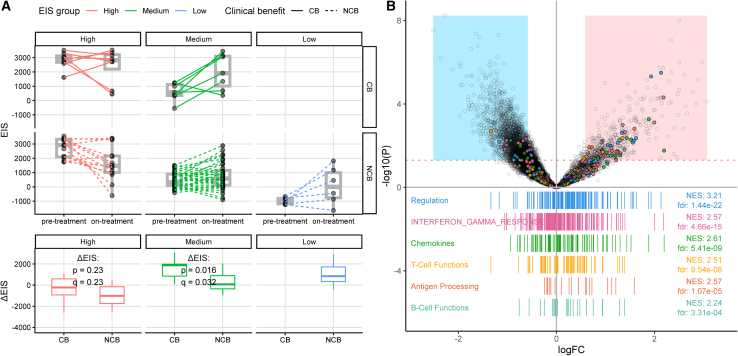
Treatment-induced transcriptional changes in patients grouped by EIS tertiles (A) Upper: paired boxplots showing pre-treatment and on-treatment EIS values for patients stratified by baseline EIS tertiles (high, medium, and low) and clinical outcome (CB or NCB). Solid and dotted lines connect paired pre-treatment and on-treatment samples from individual CB and NCB patients, respectively. Lower: per-patient ΔEIS (on-treatment minus pre-treatment) distributions comparing CB and NCB within each baseline EIS tertile; *p* values (Wilcoxon rank-sum test) and *q* values are shown for between-group comparisons. (B) Upregulated gene transcription programs associated with CB in the medium (EIS) group. Differential gene expression analysis was performed on the delta (on-treatment − pre-treatment) expression profiles. 517 CB-associated and 2,470 NCB-associated genes (|log2 fold change| > log2(1.5), *p* value < 0.05) are indicated by red and blue shaded rectangles, respectively. Enrichment of gene sets with normalized enrichment score (NES) and *q* value is shown below. See also Figure S4.

Tumors are often categorized as cold (low immune infiltration) or hot (high immune infiltration); “hot” tumors are typically more amenable to treatment with ICIs.^2^^,^^10^ Thus, we separated the pre-treatment samples into ESTIMATE immune score (EIS) tertiles (low, medium, or high; Figure 4A). All patients with tumors with low baseline EIS had NCB, regardless of the magnitude increase in EIS during treatment, whereas patients with high baseline EIS had a higher rate of CB regardless of the magnitude of decline in EIS during treatment. Notably, patients with medium EIS who achieved CB had a greater increase in EIS during treatment (*p* = 0.016, Wilcoxon rank-sum test; Figure 4A). This association was robust across multiple sensitivity analyses. Bootstrap resampling and permutation testing supported a positive ΔEIS difference between CB and NCB patients (95.4% of resamples >0; permutation *p* = 4 × 10^−4^; Figure S4A). In mixed-effects models accounting for tumor type, greater EIS increases remained associated with CB (OR: 3.60 per SD; *p* = 0.013), with consistent directionality in leave-one-tumor-type-out analyses (Figure S4B). Gene set enrichment analysis of on-treatment samples from patients who achieved CB in this group showed a marked increase in IFN-γ response, antigen processing, B cell function, and T cell regulation compared with the pre-treatment samples (Figure 4B). These data indicate that tumors with medium baseline EIS possess distinct biological features marked by treatment-induced immune activation that distinguish CB from non-benefit following pembrolizumab.

### Spatial immune characterization by multiplex immunofluorescence

**Figure 5:**
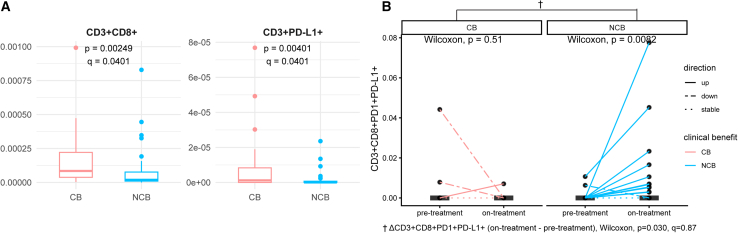
Multiplex immunofluorescence features related to clinical outcome (A) Pre-treatment densities of cytotoxic T cell (CD3^+^CD8^+^) and PD-L1-expressing T cell (CD3^+^PD-L1^+^) populations in tumors from patients with CB and NCB of pembrolizumab. Comparisons between CB and NCB were performed using the Wilcoxon rank-sum test, with *p* values adjusted for multiple testing using the false discovery rate (FDR) method. (B) On-treatment increase/decrease in the density of cytotoxic T cells expressing both PD-1 and PD-L1 in patients with CB and NCB. Lines connect paired samples within individual patients. The change in density (on-treatment minus pre-treatment) was compared between CB and NCB patients using the Wilcoxon rank-sum test and corrected for multiple testing using the FDR method. See also Figure S5.

We next performed multiplex immunofluorescence to better delineate the immune microenvironment of each tumor and characterize features related to clinical outcome. Pre-treatment tumors from patients who achieved CB showed significantly (*q* < 0.1) higher densities of CD3^+^ and CD8^+^ T cells, including proliferative (Ki67^+^) and checkpoint-engaged (PD-1^+^ and/or PD-L1^+^) subsets, compared with non-benefiting tumors (Figure 5A; Figure S5). In contrast, limited coordinated immune remodeling was observed between CB and NCB patients; only one population, CD3^+^CD8^+^PD-1^+^PD-L1^+^ T cells, showed a nominally significant (*p* = 0.030, *q* = 0.87, Wilcoxon rank-sum test) increase following treatment, preferentially in NCB patients (Figure 5B).

## Discussion

In our phase 2 study, we found that pembrolizumab elicited objective response in 14.8% and CB in 26.8% of a heterogeneous cohort of rare tumor types.^6^ Notably, CB was observed in most cancer types evaluated, with the exception of small-cell malignancies of non-pulmonary origin and medullary renal cell carcinoma, which exhibited no evidence of clinical response. These data highlight both the potential and limitations of immune checkpoint blockade in this under-studied population.

In the current study, we sought to identify potential biomarkers of response to treatment with pembrolizumab in patients with advanced rare cancers. Initially, we assessed the known markers. Because TMB-H^5^ and MSI-H^4^ tumors are susceptible to immune checkpoint blockade due to increased neoantigen load, pembrolizumab is FDA approved for TMB-H and MSI-H solid cancers. Therefore, clinically testing rare tumors for MSI or TMB provides the opportunity to administer anti-PD-1 treatment to patients regardless of the tumor type. However, in the current study, only a small fraction of rare tumors had the MSI-H (8.8%) or TMB-H status (6.2%) currently required to meet the criteria for standard-of-care pembrolizumab treatment, even though 26.8% of patients achieved CB from treatment.

In our study, patients with MSI-H or TMB-H tumors were significantly more likely to experience CB (OR: 13.9, *p* = 0.0013), aligning with established data in more common cancer types. Although these markers may serve as actionable indicators even within rare cancers, the predictive value of TMB and MSI status varies significantly across tumor types. As seen in our study, not all patients with TMB-H or MSI-H respond to treatment with ICIs. In a recent study^11^ that analyzed somatic mutation data from the Genomics Evidence Neoplasia Information Exchange (*n* = 46,320) across 22 different cancer types, a significant proportion of patients with MSI-H had a TMB-low phenotype. Significant differences in progression-free and overall survival were reported among groups of patients with differing MSI/TMB status (MSI-H and TMB-H, MSI-H and TMB-low, non-MSI-H and TMB-H, and non-MSI-H and TMB-low), suggesting distinct immune responses even within patients with MSI-H or TMB-H tumors. These findings indicate that factors beyond these DNA-based features play an important role in anti-PD-1 treatment outcomes and warrant further investigation.

We also evaluated the relationship between pre-treatment PD-L1 expression and CB. Consistent with previous evidence that patients with tumors expressing higher levels of PD-L1 had improved treatment outcomes with ICIs,^12^^,^^13^ in the current study, a combined positive score cutoff of ≥10 was linked to higher odds of achieving CB overall (OR: 2.5, *p* = 0.0285). However, this association was not consistent across tumor types (Figure 1B). This variability underscores the complexity of PD-L1 expression as a predictive biomarker.

We analyzed the gene expression profiles in pre- and on-treatment tumor tissue samples and found distinct immune landscapes between CB and NCB patients. In patients who achieved CB, genes associated with T cell activation pathways, such as those related to T cell cytotoxicity (e.g., HLA-A, GZMA/B, and PRF1) and chemokines (e.g., CXCL9, CXCL10, and CXCL13), were upregulated. In contrast, in tumors from patients with NCB, genes related to cell cycle regulation and proliferation (e.g., E2F, G2M, and MYC) as well as pathways associated with hypoxia and DNA damage repair were enriched. These observations indicate that tumors responsive to pembrolizumab exhibit more functionally antitumor immune responses in which the mechanisms of lymphocyte recruitment and cancer cell detection and targeting are relatively more intact. Conversely, tumors associated with NCB show features of unchecked proliferation and increased metabolic or cellular stress. Persistence of this dichotomy in on-treatment samples suggest that immune “rescue” in immune-excluded tumors is limited.

The tumor microenvironment is crucial in shaping the immune response to immune checkpoint blockade. Patients with lower levels of tumor immune infiltration were less likely to have CB, whereas those with higher immune infiltration were more likely to have CB. Therefore, we evaluated the pre-treatment and on-treatment levels of immune cell infiltration in the tumor microenvironment. We further stratified tumors by their pre-treatment immune infiltration using the EIS. Consistent with prior anti-PD-1/anti-PD-L1 treatment studies, CB in the current study was associated with the level of pre-treatment immune infiltration.^14^ Patients with tumors in the lowest tertile of pre-treatment immune infiltration uniformly had NCB, whereas those with tumors in the highest tertile of pre-treatment immune infiltration predominantly experienced CB regardless of decreased or increased immune infiltration. Notably, patients with intermediate-tertile immune-infiltrated tumors could still achieve CB, provided the immune content of the tumor increased during treatment. These findings highlight the importance of a pre-existing, immune-enriched tumor microenvironment as a key determinant of response and suggest that a potentially actionable subset of patients with immunologically “primed” tumors may respond to treatment with ICIs in this patient population.

To have a deeper understanding of the immune cell landscape that characterizes the tumor microenvironment in rare cancers and the dynamics of this landscape, we performed multiplex immunofluorescence studies on pre-treatment and on-treatment tumor tissue samples. At baseline, tumors from patients who achieved CB exhibited higher densities of CD3^+^ and CD8^+^ T cells, including proliferative (Ki67^+^) and checkpoint-engaged (PD-1^+^ and/or PD-L1^+^) subsets, in line with observations that productive responses depend on pre-existing antigen-experienced T cells and immune-inflamed tumor microenvironment.^15^ Among the populations evaluated for treatment-induced change, CD3^+^CD8^+^PD-1^+^PD-L1^+^ T cells showed a nominally significant on-treatment increase preferentially in non-benefiting tumors. This observation is consistent with prior reports that immune checkpoint blockade can amplify checkpoint-engaged T cell states and counter-regulatory feedback in settings where antitumor immunity is ineffective, a phenomenon often described as adaptive immune resistance.^15^^,^^16^^,^^17^

### Limitations of the study

The small sample size for each cancer type and the heterogeneity observed within the “other rare histologic type” group hinder the ability to draw generalized conclusions. The study was not powered to validate predictors of response. Although external validation in a fully independent cohort would be desirable, it is constrained by the rarity and histologic diversity of the tumor types included in this study. Identifying an appropriate population even within publicly available databases is inherently challenging in the context of rare cancers as most patients with rare cancers are neither MSI-H nor TMB-H and therefore are unlikely to receive ICIs outside of a clinical trial setting. However, to address this limitation, we performed a series of internal robustness and sensitivity analyses to evaluate the stability and generalizability of the observed immune score-based associations (Figures S3A and S4A).

We further recognize the substantial tumor heterogeneity inherent to rare cancer cohorts and the potential for bias arising from sparse tumor-specific strata. To mitigate these effects, we employed tumor-type-adjusted mixed-effects logistic regression models treating histology as a random effect, thereby accounting for between-tumor variability while estimating global immune-response associations. In addition, leave-one-tumor-type-out sensitivity analyses were conducted to assess whether results were driven by any single histologic subtype (Figures S3B and S4B).

Nevertheless, given the challenges associated with conducting clinical trials in patients with rare cancers and obtaining longitudinal samples, our single-center study serves as a valuable hypothesis-generating effort, demonstrating that immune responsiveness in rare cancers may be characterized by biological features beyond currently approved pan-cancer biomarkers, thereby providing insightful perspectives into potential predictors of response to treatment with pembrolizumab in this patient population. Our next objective is to conduct a large multi-center clinical trial that will include a greater number of patients across rare tumors subtypes, which will substantially increase cohort sizes, expand opportunities for biospecimen collection, and allow validation of the current findings.

In conclusion, the current study highlights the critical role of pre-existing immune components in mediating clinical responses to anti-PD-1 therapy in rare tumors. Our findings also emphasize the capacity of patients with intermediate immune scores to recruit additional immune cells into the tumor microenvironment. However, further investigation with a larger and more homogeneous patient cohort is essential to more precisely define the mechanisms underlying immune activation in rare tumor types.

## Resource availability

### Lead contact

Further information and requests for resources and reagents should be directed to and will be fulfilled by the lead contact, Aung Naing (anaing@mdanderson.org).

### Materials availability

This study did not generate new unique reagents.

### Data and code availability

• The sequencing data, mutation calls and expression matrix generated in this study, and relevant non-identifiable clinical metadata have been deposited in the European Genome-Phenome Archive (EGA: EGAD50000002182). Because the data contain patient-derived genomic and clinical information, access is granted in accordance with the study consent and applicable regulations. Researchers can request access by submitting a data access proposal through the EGA portal, which will be reviewed by the Data Access Committee (DAC: EGAC50000000860). Approved users will be granted access under data use agreements consistent with the study consent and governance framework.
• Code has been deposited at GitHub at https://github.com/WoodmanLab/pembro_codebase and is publicly available as of the date of publication.
• Any additional information required to reanalyze the data reported in this paper is available from the lead contact upon request.

## Acknowledgments

Merck Sharp & Dohme Corp., a subsidiary of Merck & Co., Inc., provided the study drug and funded the study. This work was supported in part by the National Cancer Institute at the National Institutes of Health (1R01CA279749-01A1 to A.N.); National Institutes of Health CCSG Award (CA016672-Institutional Tissue Bank [ITB] and Research Histology Core Laboratory [RHCL]); Adaptive Patient-Oriented Longitudinal Learning and Optimization (APOLLO) Moonshot Program, Strategic Alliances and the Translational Molecular Pathology-Immunoprofiling lab (TMP-IL) MoonShots Platform at the Department Translational Molecular Pathology, The University of Texas MD Anderson Cancer Center; National Center for Advancing Translational Sciences at the National Institutes of Health through UT Health-CCTS grant (1UM1 TR0045906); and The University of Texas MD Anderson Cancer Center through the Molecular Evaluation and/or Biopsy Related Support Program (to A.N.). We express our gratitude to the patients, their families, and caregivers for participating in the study. We thank Erica Goodoff, Senior Scientific Editor in the Research Medical Library at The University of Texas MD Anderson Cancer Center, for her expert editorial assistance in preparing this manuscript.

## Author contributions

Conceptualization, A.N., J.H., I.I.W., K.R.S., J.W., S.E.W., and F.M.-B.; data curation, X.L., M.H.D., B.S., M.X., A.A., and A.Z.; formal analysis, X.L., Q.W., X.T., M.H.D., B.S., M.X., R.K.P., M.R., E.R.P., J.S., M.M.M., J.W., and S.E.W.; funding acquisition, A.N. and K.R.S.; investigation, A.N., S.S., P.R.B., Y.Y., H.A.M., G.S., J.R.A., S.F., V.S., S.A.P.-P., and F.M.-B.; methodology, A.N., X.L., Q.W., X.T., R.K.P., M.R., E.R.P., J.S., M.M.M., J.W., and S.E.W.; project administration, A.N. and S.A.G.; resources, A.N., S.S., P.R.B., I.I.W., J.R.A., S.F., V.S., S.A.P.-P., J.W., S.E.W., and F.M.-B.; software, X.L., Q.W., X.T., S.S., P.R.B., K.R.S., R.K.P., M.R., E.R.P., J.S., M.M.M., J.W., and S.E.W.; supervision, A.N., Y.Y., H.A.M., I.I.W., K.R.S., J.W., S.E.W., and F.M.-B.; visualization, A.N., X.L., Q.W., X.T., B.S., and S.E.W.; writing – original draft, A.N., X.L., B.S., M.X., and S.E.W.; writing – review and editing, all authors. All authors had resources to access the data in the study and approved the final version of the manuscript to be published. All listed authors meet authorship criteria, and no other individuals meeting the criteria have been omitted.

## Declaration of interests

A.N. reports research funding to the institution from NCI, EMD Serono, MedImmune, Healios Inc. Nutrition, Atterocor/Millendo, Amplimmune, ARMO BioSciences, Karyopharm Therapeutics, Incyte, Novartis, Regeneron, Merck, Bristol Myers Squibb, Pfizer, CytomX Therapeutics, Neon Therapeutics, Calithera Biosciences, TopAlliance Biosciences, Eli Lilly, Kymab, PsiOxus, Arcus Biosciences, NeoImmuneTech, Immune-Onc Therapeutics, Surface Oncology, Monopteros Therapeutics, BioNTech SE, Seven & Eight Biopharma, and SOTIO Biotech AG; advisory board/consulting fees from CTI, Deka Biosciences, Janssen Biotech, NGM Bio, PsiOxus Therapeutics, Immune-Onc Therapeutics, STCube Pharmaceuticals, OncoSec KEYNOTE-695, Genome & Company, CytomX Therapeutics, Nouscom, Merck Sharp & Dohme Corp, Servier, Lynx Health, and AbbVie; travel and accommodation expense from ARMO BioSciences, NeoImmuneTech, and NGM Biopharmaceuticals; and honoraria for speaking engagements from AKH Inc., The Lynx Group, Society for Immunotherapy of Cancer (SITC), Korean Society of Medical Oncology (KSMO), Scripps Cancer Care Symposium, ASCO Direct Oncology Highlights, European Society for Medical Oncology (ESMO), and CME Outfitters.

J.H. received research funding from Immune Deficiency Foundation, Jeffrey Modell Foundation, and Takeda; served on the advisory board of Immune Deficiency Foundation and Pharming; and was a member of the speaker bureau of Pharming.

J.R.A. received research funding from Merus, Blueprint Medicines, Merck Sharp & Dohme, and 280 Bio; consulting fees from Cancer Core Europe, Hummingbird Yingli, Meru, Aadi Bioscience, ForeBio, Amgen, Monte Rosa, Debio, Incyte, BridgeBio Pharma, and Vall d’Hebron Institute of Oncology/Cancer Core Europe; and other financial or non-financial interests from Cancer Core Europe, Symphogen, BioAlta, Pfizer, Kelun-Biotech, GlaxoSmithKline, Taiho, Roche Pharmaceuticals, Hummingbird, Yingli, Bicycle Therapeutics, Merus, Aadi Bioscience, ForeBio, Loxo Oncology, Hutchinson MediPharma, Ideaya, Amgen, Tango Therapeutics, Mirati, Linnaeus Therapeutics, MonteRosa Kinnate, Debio, BioTheryX, Storm Therapeutic, BeiGene, MapKure, Relay, Novartis FusionPharma, C4 Therapeutics, Scorpion Therapeutics, Incyte, Fog Pharmaceuticals, Tyra, Nuvectis Pharma, BridgBbio Pharma, 3H Pharmaceuticals, Hummingbird, AstraZeneca, Yingli, and Vall d’Hebron Institute of Oncology/Cancer Core Europe.

S.F. received clinical trial research support/grant funding through the institution from NIH/10.13039/100000054NCI P30CA016672 – Core Grant (CCSG Shared Resources); Abbisko; Antengene; BeiGene; BeyongSpring Pharmaceuticals, Inc., LLC; Biotheus Inc.; Boehringer Ingelheim; Coherent Biopharma, LLC; Crossignal Therapeutics, Inc.; CUE Biopharma, Inc.; DEKA Biosciences; Eli Lilly & Co.; Exelixis; Fore Biotherapeutics; Greenfire Bio, Inc.; Hookipa Biotech; IMV, Inc.; Innovent Biologics, Co., Ltd.; Jazz Pharmaceuticals, K-Group Beta; Lantern Pharma Inc.; Lyvgen Biopharm, Co., Ltd.; MacroGenics; MediLink Therapeutics, Co. Ltd.; Millennium Pharmaceuticals, Inc.; Nerviano Medical Sciences; NeuPharma, Inc.; NextCure, Inc.; Ningbo NewBay Technology Development Co., Ltd.; Novartis; NovoCure; Nykode Therapeutics AS; Parexel International, LLC; PharmaMar USA, Inc.; Pionyr Immunotherapeutics, Inc.; PureTech Health, LLC; Qurgen, Inc.; Shanghai Huaota Biopharmaceutical Co., Ltd.; Sellas Life Sciences Group; Soricimed Biopharma, Inc.; SQZ Biotechnologies; Sumitomo Dainippon; Taiho Oncology and NCCN; Tigermed Group, LLC; TransCode Therapeutics, Inc.; Treadwell Therapeutics; Turnstone Biologics; Tyligand Bioscience, Ltd.; Virogin Biotech, Ltd.; and Cancer Prevention Research Institute of Texas (CPRIT) Precision Oncology Decision Support Core (RP150535).

V.S. reports research funding to the institution from AbbVie, Agensys, Alfasigma, Altum, Amgen, Arvinas, Bayer, BERG Health, Blueprint Medicine, Boston Biomedical, Boston Pharmaceuticals, Celgene, D3 Bio, Deciphera, Dragonfly Therapeutics, Exelixis, Fujifilm, GlaxoSmithKline, Idera Pharmaceuticals, Incyte, Inhibrix, Loxo Oncology, MedImmune, MultiVir, NanoCarrier, National Comprehensive Cancer Network, NCI-CTEP, Northwest Biotherapeutics, Novartis, PharmaMar, Pfizer, OnCusp, Relay Therapeutics, Roche/Genentech, Synnovation Therapeutics, Takeda, Turning Point Therapeutics, UT MD Anderson Cancer Center, Vegenics, and Xencor; and consulting advisory role payments to the institution from AbbVie, Agenus, Astex Pharmaceuticals, AstraZeneca, Bayer, Bristol Myers Squibb, Clasp Therapeutics, Clinical Care Communications, Endeavor BioMedicine, Enlaza Therapeutics, Genmab, Hyku Biosciences, Incyte, Jazz Pharmaceuticals, LabGenius Limited, Lilly, Loxo Oncology, Merck, Nimbus Discovery, Novartis, Obsidian Therapeutics, Ottimo Pharma, PERS, Pfizer, Pheon Therapeutics, Regeneron, Relay Therapeutics, Revolution Medicine, Rigel Pharmaceuticals, and Roche.

S.A.P.-P. received clinical trial research support/grant funding through the institution from ABM Therapeutics, Inc.; Alkermes; Aminex Therapeutics; BioMarin Pharmaceutical, Inc.; Boehringer Ingelheim; Chugai Pharmaceutical Co., Ltd.; Cyclacel Pharmaceuticals; Daiichi Sankyo; ENB Therapeutics; Epigenetix Inc.; Genmab US, Inc.; Gilead Sciences, Inc.; Hengrui Pharmaceuticals, Co., Ltd.; Immunity Bio, Inc.; Immunome, Inc.; Immunomedics, Inc.; Incyte Corp.; Innovent Biologics Co., Ltd.; iTeos Belgium SA; Jazz Pharmaceuticals; Johnson & Johnson; Loxo Oncology, Inc.; Merck Sharp and Dohme Corp.; Mitsubishi Tanabe Pharma America (MTPA) Inc.; Nectin Therapeutics, Ltd.; Nested Therapeutics, Inc.; NRG Oncology; Nurix; OncoNano Medicine, Inc.; Pieris Pharmaceuticals, Inc.; Pfizer; Phanes Therapeutics; Puma Biotechnology, Inc.; Purinomia Biotech, Inc.; Replimune; Roche/Blueprint; Solve Therapeutics; Strand Therapeutics, Inc.; Tallac Therapeutics, Inc.; Toragen Therapeutics, Inc.; TransThera Bio; Xencor, Inc.; NCI/NIH P30CA016672 Core Grant (CCSG Shared Resources); CPRIT Grant: CPRIT Precision Oncology Decision Support Core (RP150535); and Clinical and Translational Science Award Grant (CTSA): 1UM1TR004906; and is a consultant for MTPA Inc.

F.M.-B. received fees for consulting from AstraZeneca Pharmaceuticals, Becton Dickinson, Calibr (a division of Scripps Research), Daiichi Sankyo, Dava Oncology, Debiopharm, EcoR1 Capital, eFFECTOR Therapeutics, Elevation Oncology, Exelixis, GT Aperion, Incyte, Jazz Pharmaceuticals, LegoChem Biosciences, Menarini Group, Molecular Templates, Protai Bio, Ribometrix, Tempus, and Zymeworks; served on advisory committee of Cybrexa Therapeutics, GO Therapeutics, Guardant Health, Harbinger Health, Kivu Biosciences, Loxo Oncology, Mersana Therapeutics, OnCusp Therapeutics, Seagen, Theratechnologies, and Zentalis Pharmaceuticals; sponsored research funding (to the institution) from Jazz Pharmaceuticals, Zymeworks, Aileron Therapeutics, Inc. AstraZeneca, Bayer Healthcare Pharmaceutical, Calithera Biosciences Inc., Curis Inc., CytomX Therapeutics Inc., Daiichi Sankyo Co. Ltd., Debiopharm International, eFFECTOR Therapeutics, Genentech Inc., Guardant Health Inc., Klus Pharma, Takeda Pharmaceutical, Novartis, Puma Biotechnology Inc., and Taiho Pharmaceutical Co., Ltd.; honoraria from Dava Oncology; and other (travel-related) from European Organisation for Research and Treatment of Cancer (EORTC), ESMO, Cholangiocarcinoma Foundation, and Dava Oncology.

## STAR★Methods

### Key resources table

**Table:** 

REAGENT or RESOURCE	SOURCE	IDENTIFIER
**Antibodies**		

Mouse monoclonal anti-PD-L1 (IHC)	Agilent Technologies	Cat#SK006; RRID:AB_2889976
Rabbit recombinant monoclonal anti-PD-1	Abcam	Cat#ab137132-100μl; RRID:AB_2894867
Rabbit monoclonal anti-PD-L1 (mIF)	Cell Signaling	Cat#13684S; RRID: AB_2687655
Rabbit monoclonal anti-CD3ε	Cell Signaling	Cat#85061S; RRID: AB_2721019
Mouse monoclonal anti-CD8	Thermo Fisher	Cat#MS-457-S; RRID:AB_61027
Rabbit monoclonal anti-FOXP3	Cell Signaling	Cat#98377S; RRID: AB_2747370
Mouse monoclonal anti-CD68	Agilent	Cat#M087601-2
Mouse monoclonal anti-Ki67	Agilent	Cat#M724001-2; RRID: AB_2631211
Mouse monoclonal anti-cytokeratin	Agilent	Cat#M351501-2; RRID: AB_2631307

**Chemicals, peptides, and recombinant proteins**		

QIAamp® DNA Mini Kit	Qiagen	Cat#51306
Quant-iT™ PicoGreen™ dsDNA Assay Kit	Thermo Fisher Scientific	Cat#P7589
Genomic DNA ScreenTape and Reagents	Agilent Technologies	Part Number:5067–5365;5067-5366
NORGEN Total RNA Purification Kit	Norgen Biotek	Cat#37500
AMPure XP beads	Beckman Coulter	Cat#A63882
Qubit™ RNA HS Assay Kit	Life Technologies	Cat#Q32855
RNA 6000 Nano Kit	Agilent Technologies	Part Number:5067-1511

**Critical commercial assays**		

Ovation® RNA-Seq System V2	NuGEN	Cat#7102-A01
SureSelect XT Low Input Reagent Kit	Agilent Technologies	Part Number:G9707A-G9707L
SureSelect Human All Exon v4	Agilent Technologies	Part Number:5190-4638
PD-L1 IHC 22C3 pharmDx	Agilent Technologies	Cat#SK006

**Deposited data**		

RNA-seq data	This paper	EGAS50000001509
Whole-exome sequencing data	This paper	EGAS50000001508
Code	This paper	https://github.com/WoodmanLab/pembro_codebase

**Software and algorithms**		

STAR	Dobin et al.^18^	https://github.com/alexdobin/STAR
BWA	Li and Durbin^19^	https://github.com/lh3/bwa
Picard	Broad Institute	https://broadinstitute.github.io/picard/
MuTect	Broad Institute	https://software.broadinstitute.org/cancer/cga/mutect
Pindel	Ye et al.^20^	http://gmt.genome.wustl.edu/packages/pindel/
featureCounts	Liao et al.^21^	http://subread.sourceforge.net
QualiMap	Okonechnikov et al.^22^	http://qualimap.conesalab.org/
limma	R/Bioconductor	https://bioconductor.org/packages/limma
fgsea	R/Bioconductor	https://bioconductor.org/packages/fgsea
immunedeconv	Sturm et al.^23^	https://cran.r-project.org/package=immunedeconv
inForm	Akoya Biosciences	Version 2.2.1
R	R Core Team	Version 4.2.2
SAS	SAS Institute	Version 9.4

**Other**		

Web-based lesion selection tool	Xu et al.^24^	Xu et al.^24^

### Experimental model and study participant details

#### Human subjects

This was a single-center, open-label, phase II study of pembrolizumab in patients with advanced rare cancers conducted at The University of Texas MD Anderson Cancer Center. Eligible patients had tumors that progressed on standard therapies within the previous 6 months. A total of 154 patients with advanced or metastatic rare cancers were enrolled into nine tumor-specific cohorts and one additional cohort for other rare histologic types (Table 1). The median age was 57 years (range, 22–88), and 53% of them were men. Most patients (89%) had ECOG performance status of 1. Prior to enrollment, patients had received a median of 2 lines of therapy (range, 0–10). Of the 154 enrolled patients, 142 were evaluable for response, of whom 38 achieved CB and 104 did not (NCB). Pre-treatment tumor samples were available for transcriptomic analysis in 89 patients (CB *n* = 19, NCB *n* = 70), with matched on-treatment samples available in a subset (*n* = 71; CB *n* = 15, NCB *n* = 56). PD-L1 assessment was performed on 124 pre-treatment samples. Multiplex immunofluorescence was performed on 86 pre-treatment samples (CB *n* = 23, NCB *n* = 63), with matched on-treatment samples available for 64 patients (CB *n* = 12, NCB *n* = 52).

Among the study population, 56% had a PD-L1 CPS < 10, and 39% had TIL score of 1. Sex was not significantly associated with CB (Fisher’s exact test: OR 0.65, 95% CI 0.28–1.48; *p* = 0.343) or multivariable logistic regression adjusting for tumor type (OR 0.52; *p* = 0.152).

Patients received pembrolizumab at a fixed dose of 200 mg intravenously every 21 days until documented disease progression, unacceptable adverse events, investigator decision to discontinue treatment, withdrawal of consent, or completion of 24 months of therapy. Tumor response was assessed every 9 weeks using immune-related Response Evaluation Criteria in Solid Tumors (irRECIST).^25^ Adverse events were graded according to the National Cancer Institute Common Terminology Criteria for Adverse Events (CTCAE) version 4.03.^26^ The study protocol was approved by the U.S. Food and Drug Administration and the Institutional Review Board (IRB) at The University of Texas MD Anderson Cancer Center. The study obtained ethics approval from the IRB of The University of Texas MD Anderson Cancer Center (Federalwide Assurance Number [FWA]: 00000363 and Office of Human Research Protection [OHRP] IRB Registration Number: IRB00000121). The study was conducted in accordance with the Declaration of Helsinki and International Conference on Harmonization Good Clinical Practice guidelines. All participants provided written informed consent prior to enrollment.

### Method details

#### Tissue and blood collection

Tumor tissue was obtained at baseline (within 28 days prior to treatment initiation), during treatment (between cycle 1 day 15 and day 21), and at progression for patients with complete response, partial response, or stable disease lasting longer than 27 weeks, provided that a tumor lesion was safely biopsiable. Exceptions were permitted for patients with paraganglioma/pheochromocytoma or other rare histologies in which biopsies were not feasible due to predominant bone disease or other clinical constraints. A web-based lesion selection tool^24^ was used to ensure consistent sampling of the same lesion across time points. When feasible, up to five biopsy cores were collected per procedure. Cores 1 and 2 were formalin-fixed and paraffin-embedded (FFPE) for multiplex immunofluorescence studies, while cores 3–5 were flash frozen in liquid nitrogen for RNA sequencing and whole-exome sequencing.

Archival tumor tissue or baseline biopsy material (if archival tissue was unavailable) was sent to a central laboratory (QualTek Molecular Laboratories) for assessment of PD-L1 expression and TILs. Peripheral blood samples were collected at baseline, during tumor restaging, concurrent with tumor biopsies, and at the time of disease progression.

#### Immunohistochemistry

##### PD-L1 assessment

PD-L1 expression was evaluated on baseline FFPE tumor sections using the PD-L1 IHC 22C3 pharmDx assay (Agilent Technologies). PD-L1 expression was reported as a CPS, defined as the percentage of PD-L1–positive tumor cells and mononuclear inflammatory cells within tumor nests and adjacent stroma. Samples with CPS ≥10 were considered PD-L1 positive.

##### Tumor-infiltrating lymphocytes

TILs within tumor nests were scored on a semi-quantitative scale from 0 to 3: 0, absence of TILs per high-power field; 1, 1–10 TILs per high-power field; 2, 11–20 TILs per high-power field; and 3, >20 TILs per high-power field.

#### Sequencing and data processing

##### DNA extraction and quality control

gDNA was extracted by QIAamp DNA Mini Kit (Qiagen) then quantified using Quant-iT PicoGreen dsDNA Assay Kit (ThermoFisher SCIENTIFIC) and quality was accessed using Genomic DNA ScreenTape and Reagents on the Tapestation 4200 (Agilent Technologies).

##### RNA extraction and quality control

RNA was extracted by NORGEN Total RNA Purification Kit (Cat. 37500) (NORGEN BIOTEK CORP). The extracted RNA was treated with DNase I to get rid of nay genomic DNA residues. The treated RNA then was cleaned-up using the AMPure XP beads (Beckman Coulter Life Sciences) and eluted into 1x TE buffer. The purified RNA was quantified using Qubit RNA HS Assay Kit (Life Technologies) and the RNA quality was accessed using Agilent RNA 6000 Nano Kit and the 2100 Bioanalyzer Instrument (Agilent Technologies).

##### cDNA synthesis

The cDNA was prepared from the extracted total RNA using Ovation RNA-Seq System V2 (NuGEN). Amplification is initiated at the 3′ end as well as randomly throughout the transcriptome in the sample. The prepared cDNA was quantified using Quant-iT PicoGreen dsDNA Assay Kit (ThermoFisher SCIENTIFIC) and quality was accessed using Genomic DNA ScreenTape and Reagents on the Tapestation 4200 (Agilent Technologies).

##### Library preparation

Up to 200 ng of each gDNA/cDNA sample based on the PicoGreen quantification was sheared (mechanically fragmented) using the E220 Focused-ultrasonicator Covaris (Covaris). The sonication was performed under the following conditions: Peak Incident Power 200, Duty Cycle 25%, Cycles per Burst 50, and duration 10 s for 70 iterations. To ensure the proper fragment size, samples were examined on TapeStation 4200 using the DNA High Sensitivity kit (Agilent Technologies). The sheared DNA was proceeded to library preparation using SureSelect XT Low Input Reagent Kit with indexes 1–96 (Agilent Technologies) as automated method on the Sciclone G3 NGSx Workstation (PerkinElmer, Inc.).

This protocol consists of 3 enzymatic reactions for end repair, A-tailing and Adaptor ligation, followed by barcode insertion by PCR using Herculase II Fusion DNA Polymerase (8–14 cycles, based on input DNA quality and quantity). PCR primers were removed by using 1x volume of Agencourt AMPure PCR Purification kit (Agencourt Bioscience Corporation). The quality and quantity of the prepared libraries were evaluated using TapeStation 4200 and the DNA High Sensitivity kit (Agilent Technologies) to verify correct fragment size and to ensure complete removal of primer dimers.

##### Hybridization and capture

The prepared libraries were individually hybridized to Agilent SureSelect Human All Exon v.4 probes (Agilent Technologies). The hybridization steps were automated on the Sciclone G3 NGSx Workstation (PerkinElmer, Inc.). Agilent captures were hybridized as single sample reactions using 500 - 1000ng of prepared library as input. All Hybridization and Post-hybridization capture & washes were performed according to Agilent’s protocol.

Briefly, the capture reagents and probes were added to the prepared libraries, the mixture was incubated at 65°C on thermocycler with heated lid on for up to 24 h. The targeted regions were captured using streptavidin beads and the streptavidin-biotin-probe-target complex was washed and the captured libraries were enriched by PCR amplification according to manufacturer’s protocol. The quality and quantity of each captured sample was analyzed on TapeStation 4200 using the DNA High Sensitivity kit.

##### Sequencing and data analysis

The captured libraries were sequenced on Illumina NovaSeq 6000 platform for 2 × 150 paired end reads with an 8nt read for indexes using Cycle Sequencing v3 reagents (Illumina). For whole exome runs, the resulting BCL files containing the sequence data were converted into “.fastq.gz” files and individual libraries within the samples were demultiplexed using CASAVA 1.8.2 with no mismatches. All regions were covered by > 20 reads. For data analysis, we aligned the WEX capture deep-sequencing data to human reference assembly hg19 using BWA^19^ and removed duplicated reads using Picard.^27^

For RNA sequencing runs, raw data was processed by an in-house RNA Seq data analysis pipeline, which, among other tools uses the STAR aligner^18^ to align raw reads to hg19 version of Human reference genome, featureCounts^21^ to quantify aligned reads with to produce raw counts, and FastQC and QualiMap^22^ to evaluate quality of raw reads and feature counts.

#### Mutation analysis

Somatic single-nucleotide variants were identified using MuTect, and short insertions and deletions were called using Pindel.^20^ Post-calling quality filters were applied to retain variants that (1) were located within the targeted sequencing regions, (2) passed a mapping quality threshold of 25, (3) showed a statistically significant difference in alternate/reference allele ratio between tumor and matched normal samples (proportion test *p* ≤ 0.05), (4) had a minimum coverage of 10× in both tumor and normal samples, and (5) passed a tumor event log likelihood threshold of 6.3.

#### Tumor mutation burden and microsatellite instability

TMB was calculated from whole-exome sequencing data using a total target region size of 50 Mb. A cutoff of ≥10 mutations per megabase was used to define TMB-high tumors. MSI status was inferred based on the presence of known pathogenic or likely pathogenic mutations in the mismatch repair genes MSH2, MSH6, MLH1, or PMS2.

#### Multiplex immunofluorescence

Multiplex immunofluorescence was performed using Opal chemistry and the Vectra multispectral microscopy systemq with an automated nine-color panel targeting PD-L1, PD-1, CD3, CD8, FOXP3, CD68, Ki67, pan-cytokeratin. FFPE tissue samples from 86 patients (13 responders and 73 non-responders) were stained, scanned using the Vectra/Polaris 3.0.3 digital pathology system, and analyzed with inForm software. Tumor regions were segmented into epithelial and stromal compartments based on pan-cytokeratin expression. Cell phenotypes were defined by marker co-expression, and cell densities were calculated as the average number of cells per square millimeter across regions of interest.

#### Differential gene expression and gene set enrichment analysis

Gene expression levels were quantified as log2-transformed transcripts per million (log2[TPM +1]). Treatment-induced expression changes were calculated as the difference between on-treatment and pre-treatment expression values and correlated with clinical outcomes.

Differential gene expression analysis was performed using the limma package in trend mode, with *p* values adjusted for multiple testing using the Benjamini–Hochberg false discovery rate method. Genes with an adjusted *p* value < 0.05 were considered differentially expressed.

Gene set enrichment analysis was conducted using the fgsea package (version 1.16.0) with default parameters. Hallmark and nanoString gene sets were used, including PanCancer Immune Profiling, PanCancer Pathways, PanCancer Progression, and Vantage RNA Cancer Metabolism gene sets. Gene sets with FDR-adjusted *p* values < 0.05 were considered significantly enriched.

#### ESTIMATE score

The ESTIMATE immune score was calculated using the immunedeconv R package (version 2.1.0) via the deconvolute_estimate function.^23^ This approach applies the ESTIMATE algorithm to infer tumor purity and stromal and immune cell infiltration based on gene expression data.

### Quantification and statistical analysis

Patient baseline characteristics were summarized using descriptive statistics. CB was defined as complete response, partial response, or stable disease lasting at least 6 months according to irRECIST criteria.

Associations between biomarkers and CB were evaluated using Chi-square or Fisher’s exact tests, as appropriate. Odds ratios were calculated to estimate the effect size of each biomarker. Continuous variables were compared between CB groups using the Wilcoxon rank-sum test.

To assess robustness of immune score–based associations and mitigate bias arising from sparse tumor-type strata, multiple sensitivity analyses were performed. Nonparametric bootstrap resampling (5,000 iterations) was used to evaluate stability of effect direction and magnitude for differences in immune score–based metrics between CB groups. Permutation testing was additionally applied where appropriate to assess significance without reliance on asymptotic assumptions. Tumor heterogeneity was further addressed using mixed-effects logistic regression models with tumor type modeled as a random intercept. Finally, leave-one–tumor-type-out analyses were conducted to evaluate whether observed associations were driven by any single histologic subtype.

All statistical analyses were performed using SAS version 9.4 and R software. Two-sided *p*-values <0.05 were considered statistically significant, where applicable; for analyses involving multiple testing, significance was assessed using FDR-adjusted q-values and q-values<0.1 were considered statistically significant.

### Additional resources

The phase II clinical trial of pembrolizumab in patients with advanced or metastatic rare cancers (NCT02721732) has been registered on https://clinicaltrials.gov/study/NCT02721732.

## Supplemental information

Document S1. Figures S1–S5 and Tables S1 and S2

Document S2. Article plus supplemental information
