# Supplementary material for: Biomarker study of pembrolizumab in patients with advanced rare cancers
Source: Cell Rep Med. 2026 Jun 16;7(6):102827. doi: 10.1016/j.xcrm.2026.102827 (PMC13293929; doi:10.1016/j.xcrm.2026.102827)
Supplement: Document S1. Figures S1–S5 and Tables S1 and S2 [file mmc1.pdf]

Cell Reports Medicine, Volume *7*

Supplemental information

Biomarker study of pembrolizumab in patients with advanced rare cancers

Aung Naing, Xiqi Li, Qi Wang, Xiangjun (Jun) Tian, Sharjeel Sabir, Priya R. Bhosale, Mohamed H. Derbala, Bettzy Stephen, Mingxuan Xu, Joud Hajjar, Serdar A. Gurses, Yali Yang, Hassan Ahmed Momin, Ignacio Ivan Wistuba, Anas Alshawa, Kenna R. Shaw, Abdulrazzak Zarifa, Gopal Singh, Renganayaki Krishna Pandurengan, Gabriela Maria Raso, Edwin Roger Parra, Juhee Song, Mohammad Moustaf Mohammad, Jordi Rodon Ahnert, Siqing Fu, Vivek Subbiah, Sarina Anne Piha-Paul, Jing Wang, Scott Eric Woodman, and Funda Meric-Bernstam

SUPPLEMENTARY INFORMATION

Supplementary Figures

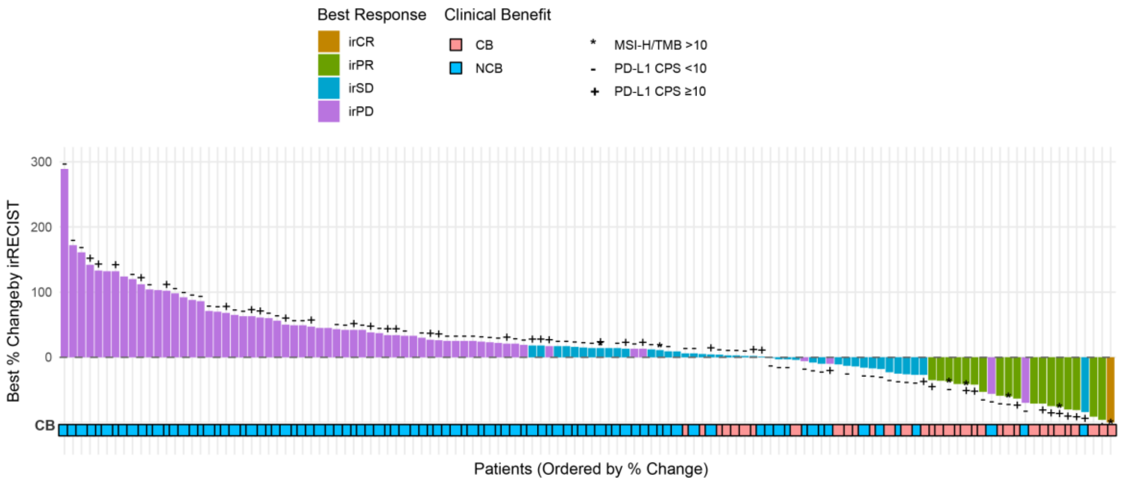

Figure S1. Waterfall plot of best response to pembrolizumab across rare cancer cohorts. Related to Table 1. Waterfall plot showing the maximum percent change from baseline in target lesion size by irRECIST for all evaluable patients treated with pembrolizumab. Each bar represents an individual patient and is colored by best overall response (irCR, irPR, irSD, irPD, or clinical progression). Bars extending below zero indicate tumor shrinkage, whereas bars extending above zero indicate tumor growth. Patients are ordered by magnitude of best response. Clinical benefit (CB) is defined as complete response, partial response, or stable disease lasting ≥6 months by irRECIST.

Abbreviations: CB, clinical benefit; CPS, combined positive score; irCR, immune-related complete response; irPR, immune-related partial response; irPD, immune-related progressive disease; irRECIST, Immune-related Response Evaluation Criteria In Solid Tumors; irSD, immune-related stable disease; MSI-H, microsatellite instability-high; NCB, no clinical benefit; PD-L1, programmed death-ligand 1; TMB, tumor mutation burden

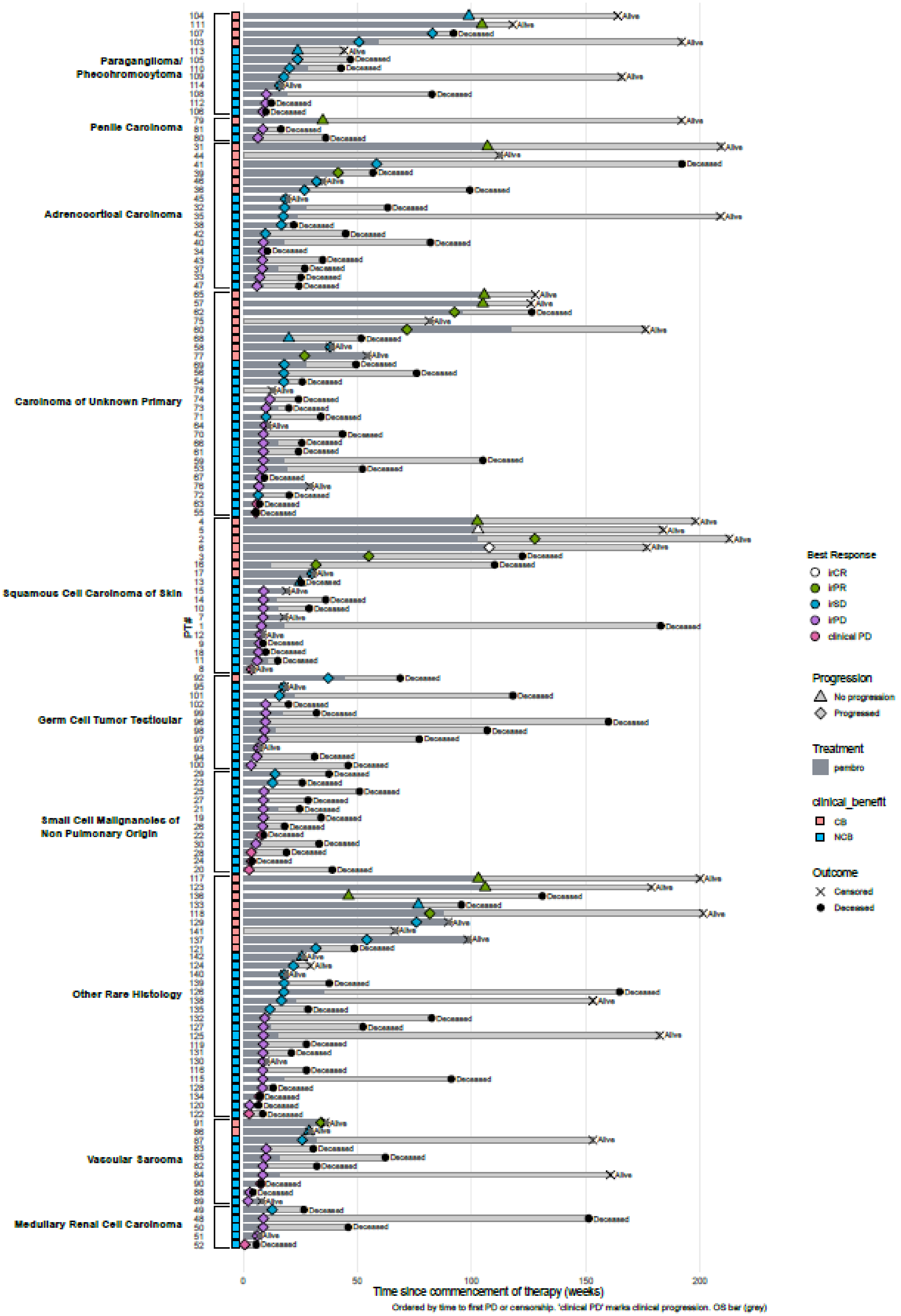

Paraganglioma/ Pheochromocytoma
Penile Carcinoma
Adrenocortical Carcinoma
Carcinoma of Unknown Primary
Squamous Cell Carcinoma of Skin
Germ Cell Tumor Testicular
Small Cell Malignancies of Non Pulmonary Origin
Other Rare Histology
Vascular Sarcoma
Medullary Renal Cell Carcinoma
PTs
0
50
100
150
200
Time since commencement of therapy (weeks)
Ordered by time to first PD or censorship. 'clinical PD' marks clinical progression. OS bar (grey)
Best Response
irCR
irPR
irSD
irPD
clinical PD
Progression
No progression
Progressed
Treatment
pembro
clinical_benefit
CB
NCB
Outcome
Censored
Deceased

Figure S2. Swimmer plot depicting treatment duration and outcomes across rare cancer types. Related to Table 1. Swimmer plot summarizing treatment duration, clinical benefit, and outcomes for all evaluable patients treated with pembrolizumab, grouped by tumor type. Each horizontal bar represents an individual patient, with bar length indicating time on therapy (weeks). Symbols denote best overall response by irRECIST (irCR, irPR, irSD, irPD, or clinical progression). Circles indicate death events, and crosses indicate censored observations at last follow-up. Patients are ordered within each tumor type by time to progression or censoring. Gray bars represent overall survival duration. Clinical benefit (CB) and no clinical benefit (NCB) status are indicated by color. Abbreviations: CB, clinical benefit; irCR, immune-related complete response; irPR, immune-related partial response; irPD, immune-related progressive disease; irRECIST, Immune-related Response Evaluation Criteria In Solid Tumors; irSD, immune-related stable disease; NCB, no clinical benefit

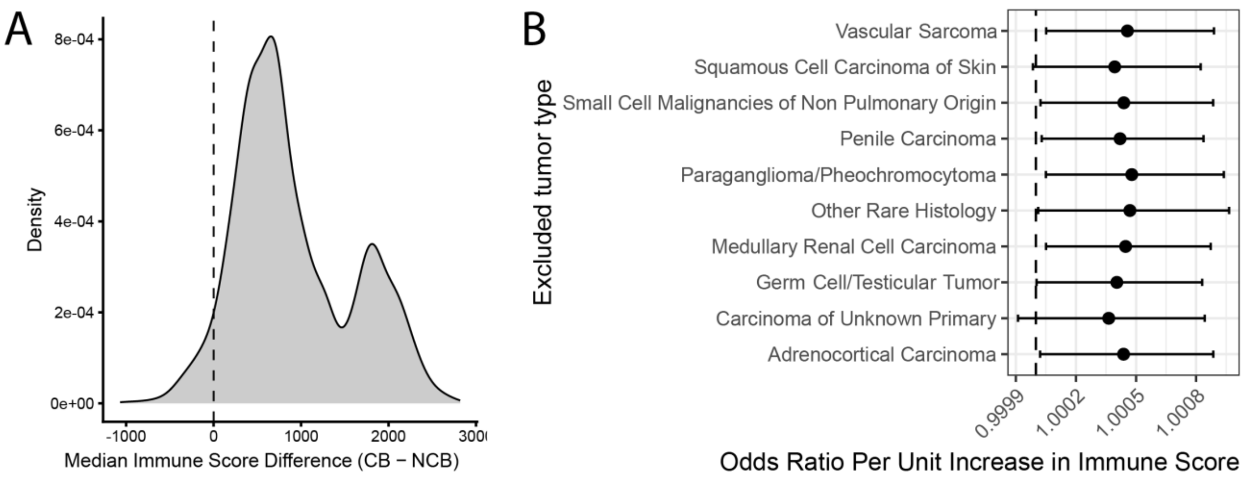

Figure S3. Robustness of pre-treatment immune score association with clinical benefit. Related to Figure 2. A. Bootstrap resampling of the difference in pre-treatment immune score between patients with clinical benefit (CB) and no clinical benefit (NCB). The distribution represents 5,000 bootstrap resamples of the median immune score difference (CB − NCB). The majority of resamples (95.2%) yielded a positive difference, indicating robust directional consistency of the observed association. **B.** Leave-one–tumor-type-out sensitivity analysis of the association between pre-treatment immune score and clinical benefit. Each point represents the odds ratio estimated after excluding one tumor type at a time; horizontal bars denote 95% confidence intervals. The dashed vertical line indicates an odds ratio of 1. Directional consistency across all exclusions indicates that the observed association is not driven by any single histologic subtype.

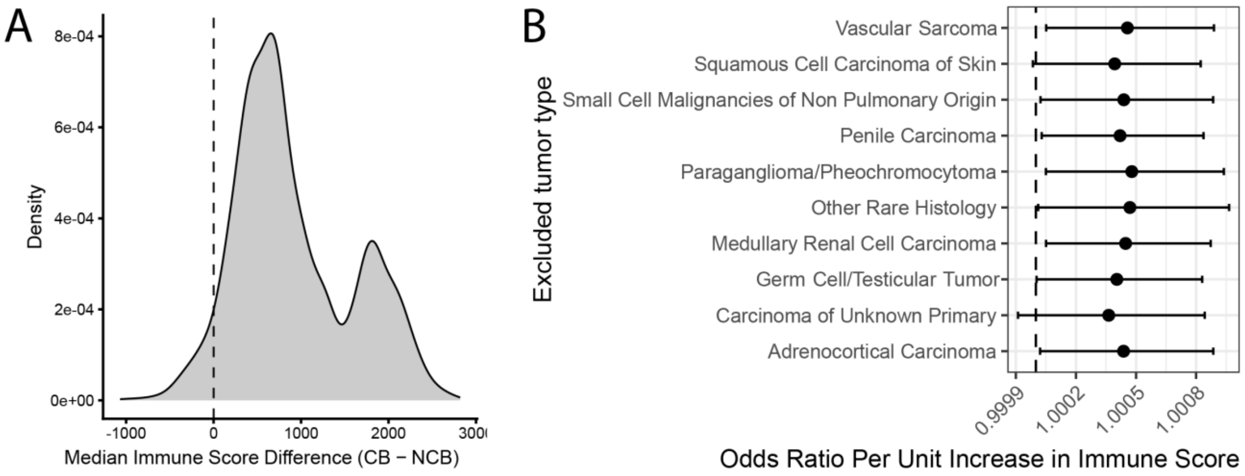

Figure S4. Robustness analyses of on-treatment changes in immune score (ΔEIS) within the medium baseline immune score subgroup. **Related to Figure 4. A.** Nonparametric bootstrap resampling (5,000 iterations) of the median ΔEIS difference between patients with clinical benefit (CB) and no clinical benefit (NCB). The distribution represents bootstrap estimates of the median difference (CB − NCB); 95.4% of resamples yielded a positive difference, indicating stable effect directionality. **B.** Leave-one–tumor-type-out sensitivity analysis of the association between ΔEIS and clinical benefit. Each point represents the odds ratio per standard deviation increase in ΔEIS estimated after excluding one tumor type at a time; horizontal bars denote 95% confidence intervals. The dashed vertical line indicates an odds ratio of 1. Consistent directionality across exclusions indicates that the observed association is not driven by any single histologic subtype.

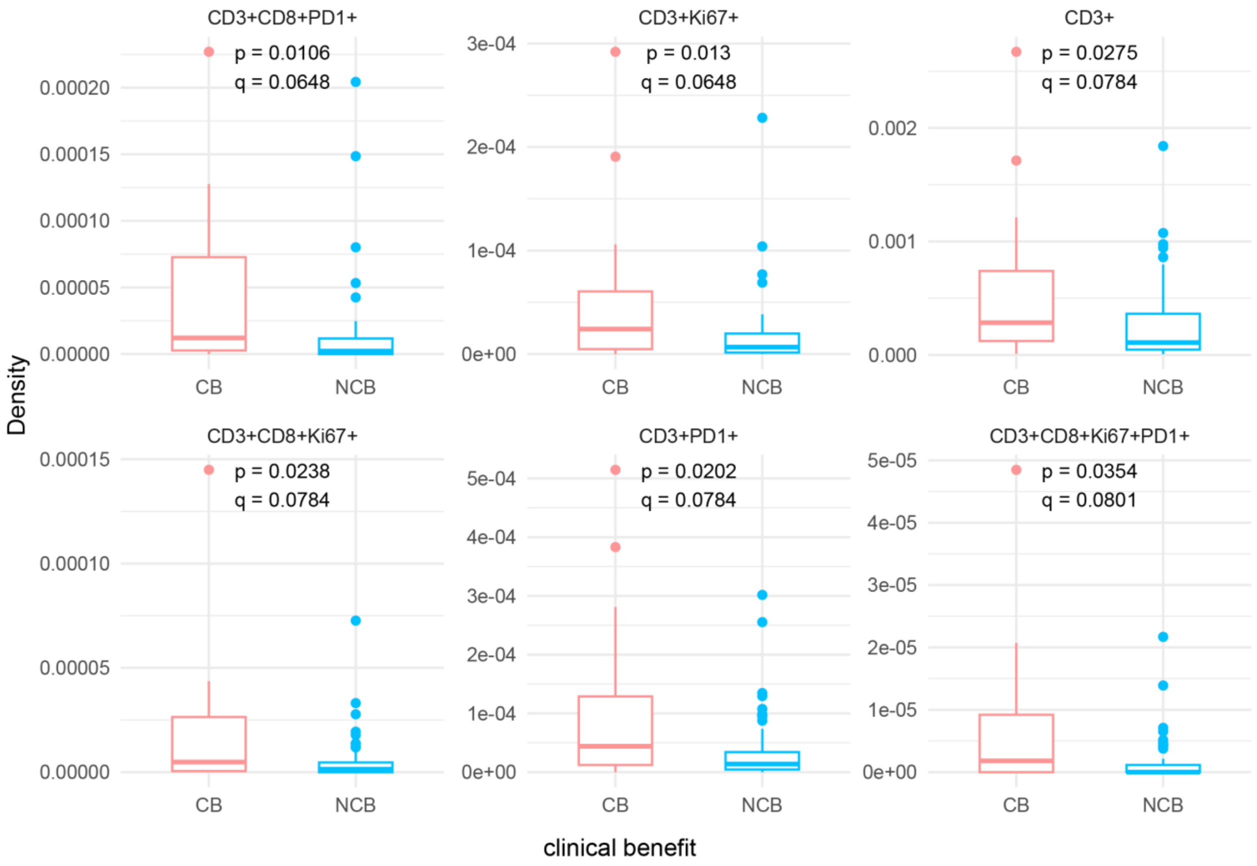

Figure S5. Pre-treatment multiplex immunofluorescence features related to clinical outcome. Related to Figure 5. Densities of selected T cell populations in tumors from patients with clinical benefit (CB) and no clinical benefit (NCB). Comparisons between CB and NCB were performed using the Wilcoxon rank-sum test, with p values corrected for multiple testing using the false discovery rate (FDR) method. Features shown met an FDR threshold of $q < 0.1$.

Supplementary Tables

Table S1. Clinical benefit by cancer type (n=142 evaluable). Related to Table 1.

Cohorts	No clinical benefit	Clinical benefit
01 Squamous cell carcinoma of the skin*	11	7
02 Small cell malignancies of non-pulmonary origin	12	0
Cervical	6	0
Desmoplastic of unknown primary	1	0
Prostate	4	0
Vulva	1	0
03 Adrenocortical carcinoma	11	6
04 Medullary renal cell carcinoma	5	0
05 Carcinoma of unknown primary	18	8
06 Penile carcinoma	2	1
07 Vascular sarcoma	8	2
Angiosarcoma	5	2
Epitheliod hemangioendothelioma	1	0
Hemangiopericytoma	2	0
08 Germ cell/testicular tumor	10	1
09 Paraganglioma/pheochromocytoma	8	4
10 Other rare histologic type	19	9
10-01 Adenoaquamous carcinoma of cervix	1	0
10-02 Adenocarcinoma of the cervix	1	0
10-03 Alveolar rhabdomyosarcoma	1	0
10-04 Alveolar soft part sarcoma	0	1
10-05 Cancer of orbit	0	1
10-06 Cranial meninges	1	0

10-07 Epitheliod neoplasm of the dermis	0	1
10-08 Granulosa cell tumor of ovary	3	2
10-09 High grade sarcoma	1	0
10-10 Liposarcoma	1	0
10-11 Malignant peripheral nerve sheath tumor	1	0
10-12 Mesothelioma in left testicle	0	1
10-13 Neuroblastoma	0	1
10-14 Pituitary	1	1
10-15 Pituitary-Expansion	1	1
10-16 Rhabdomyosarcoma	1	0
10-17 SCC of vagina	2	0
10-18 SCC of vagina-Expansion	1	0
10-19 Sclerosing epitheliod fibrosarcoma	1	0
10-20 Uterine inflammatory myofibroblastic tumor (STUMP)	1	0
10-21 Wilms tumor	1	0

* All individuals in this cohort have metastatic disease.Abbreviation: SCC, squamous cell carcinoma

Table S2. Clinical benefit by approved biomarkers (microsatellite instability and tumor mutation burden). Related to Figure 1A

Response	MSI status	TMB	Cohorts
CB	MSI-H	TMB-H	10 Other rare histologic type
CB	MSI-H	TMB-H	01 Squamous cell carcinoma of skin
CB	MSI-H	TMB-H	05 Carcinoma of unknown primary
CB	MSS	TMB-H	01 Squamous cell carcinoma of skin
CB	NA	TMB-H	05 Carcinoma of unknown primary
CB	MSI-H	NA	09 Paraganglioma/pheochromocytoma
NCB	MSS	TMB-H	10 Other rare histologic type
NCB	MSI-H	TMB-L	03 Adrenocortical carcinoma

Abbreviations: CB, clinical benefit; MSI, Microsatellite instability; MSI-H, Microsatellite instability-high; MSS, Microsatellite stable; NCB, no clinical benefit; NA, not available; TMB, Tumor mutational burden; TMB-H, Tumor mutational burden-high; TMB-L, Tumor mutational burden-low
